# A Fluorescent Lateral Flow Immunoassay for the Detection of Skeletal Muscle Troponin I in Serum for Muscle Injury Monitoring at the Point of Care

**DOI:** 10.3390/bios14080381

**Published:** 2024-08-08

**Authors:** Deding Tang, Shuang Wu, Mengqi Kong, Zhaonan Liu, Zonghao Li, Ying Han, Yan Gong, Jie Hu

**Affiliations:** 1 The Key Laboratory of Biomedical Information Engineering of Ministry of Education, School of Life Science and Technology, Xi’an Jiaotong University, Xi’an 710049, China; 10085@massz.edu.cn (D.T.);; 2 Bioinspired Engineering and Biomechanics Center (BEBC), Xi’an Jiaotong University, Xi’an 710049, China; 3 Public Teaching Department, Maanshan Teacher’s College, Maanshan 243041, China; 4 Suzhou Diyinan Biotech Company, Suzhou 215129, China; 5 School of Chemistry and Life Sciences, Suzhou University of Science and Technology, Suzhou 215009, China

**Keywords:** lateral flow immunoassay, sports medicine, exercise-induced muscle injury, point-of-care testing

## Abstract

Exercise-induced muscle injury is one of the most common types of sports injuries. Skeletal muscle troponin I (skTnI) serves as an ideal biomarker in assessing such injuries, facilitating timely detection and evaluation. In this study, we develop a fluorescent sandwich lateral flow immunoassay (LFIA) combined with a desktop analyzer for rapid detection of skTnI. Through optimizing the reaction system, the assay achieves a satisfying detection performance, reaching a limit of detection (LOD) of 0.5 ng/mL with a turnaround time of 15 min. The proposed detection platform offers portability, ease of use, and high sensitivity, which facilitates the monitoring of exercise-induced muscle injuries at the point of care. This feature is particularly advantageous for end users, enabling timely detection of sports-related injuries and ultimately enhancing prognosis and sports life.

## 1. Introduction

Life lies in exercise. Insufficient physical activity impairs people’s current and future health; however, unaccustomed or excessive physical exercise may lead to muscle injuries [1,2,3]. The predominant type of injury is skeletal muscle injury, typically induced by direct or indirect trauma to muscle soft tissues, such as contusions, strains, or tears [4]. These incidents cause alterations in the microstructure of muscle fibers, resulting in delayed-onset muscle soreness and decreased maximum voluntary contraction strength, thereby impairing motor function [5]. In severe instances, individuals may experience localized scarring, complete loss of muscle function, and notable muscle structure atrophy [6]. Due to the prolonged healing time of skeletal muscle injuries, it is usually unattainable to fully recover, and local pain and stiffness may still exist [7]. This restricts the maintenance and development of individual sport skills and adversely affects daily life and the ability to continue exercising [8]. Therefore, from the perspective of sports medicine, timely detection and efficient management of muscle injuries are crucial for improving the physical fitness of athletes, reducing the risk of sports injuries, and prolonging their sport life span [8,9].

At present, conventional detection of muscle injuries mainly relies on biochemical and electromyographic indicators [3,10]. However, traditional electromyography equipment lacks portability and usually requires testing in laboratories, leading to inconvenient access and time-consuming procedures. In addition, these traditional methods lack timely and effective monitoring and intervention capabilities, which makes them challenging in identifying early muscle injures [11]. Therefore, there is an urgent need to develop a convenient, low-cost, and user-friendly method for detecting biochemical indicators of muscle injury.

Emerging methods that rely on the detection of biomarkers from body fluids (such as the detection of creatine kinase (CK) and myoglobin (Myo)) provide a rapid and easy-to-implement approach. These methods are particularly valuable for indicating muscle injuries at an early stage and for monitoring disease progression and treatment response. Skeletal muscle troponin I (skTnI) emerges as an ideal biomarker due to its high specificity, high sensitivity and broad diagnostic window for skeletal muscle injuries [12,13]. During muscle injury, skTnI is released from damaged muscle cells into the bloodstream [1]. Currently, there are no clinical guidelines for skTnI diagnosis. By carefully checking the relative reports, the median skTnI concentrations were approximately several ng/mL in healthy reference individuals [14], and lower than 100 ng/mL in both patients with orthopedic and soft tissue injuries [15] and healthy young athletes after high-force eccentric exercise [13].

By measuring the serum skTnI concentration changes, the degree of muscle fiber damage can be assessed to facilitate timely injury detection and severity evaluation. Based on changes in skTnI concentration, medical professionals can devise personalized treatment strategies to help patients recover. However, these tests currently rely on central laboratories, requiring professional personnel and complex equipment. Thus, there is an urgent need to develop a convenient, low-cost, and user-friendly point-of-care testing method for detecting the skTnl of muscle injury.

LFIA, a well-established and widely employed point-of-care detection technology, provides advantages such as simple operation, low cost, and easy result readout interpretation [16,17]. However, compared to traditional detection methods such as enzyme-linked immunosorbent assay (ELISA) and chemiluminescence, this method typically exhibits lower sensitivity. To overcome this limitation, we developed a lateral flow assay device for rapid and highly sensitive detection of skTnI, enabling immediate assessment and prolonged monitoring of post-exercise muscle injuries. This detection platform helps athletes to promptly identify exercise-induced injuries, while improving prognosis and treatment efficacy, and ultimately prolong their overall athletic lifespan. To the best of our knowledge, this is the first study to develop a LFIA for monitoring of skTnI at the point of care.

## 2. Materials and Methods

### 2.1. Materials

Europium-chelate polystyrene microsphere (EuPSM) was purchased from VDO Biotech Co., Ltd. (Suzhou, China). Trizma base (Tris), 1-ethyl-3-(3-dimethylaminopropyL)-carbodiimide hydrochloride (EDC) and bovine serum albumin (BSA) were purchased from Sigma Aldrich (St. Louis, MO, USA), and N-hydroxysulfosuccinimide sodium salt (Sulfo-NHS) and glycine (Gly) were obtained from ShanghaiAladdin Bio-Chem Technology Co., Ltd. (Shanghai, China). Ethanolamine, ethylenediamine tetraacetic acid disodium salt dihydrate (EDTA), sodium dihydrogen phosphate dihydrate (NaH_2_PO_4_•2H_2_O), disodium hydrogen phosphate (Na_2_HPO_4_) and sodium chloride (NaCl) were obtained from China National Medicines Corporation Ltd. (Shanghai, China). MES monohydrate and Hepes were purchased from Beijing Solarbio Science & Technology Co., Ltd. (Beijing, China). Tween 20 and d-trehalose were obtained from Shanghai Macklin Biochemical Technology Co., Ltd. (Shanghai, China). Surfactant S9 was obtained from Jiangsu Pengxin Biochemical Co., Ltd. (Nantong, China). Antibodies (anti-skTnI Ab1 (5.3 mg/mL), anti-skTnI Ab2 (5.5 mg/mL)) and skTnI antigen were purchased from Hytest Ltd. (Turku, Finland). Antibodies (DNP-BSA) and DNP were purchased from Hangzhou Bioeast Biotech Co., Ltd. (Hangzhou, China). A backing pad (80 × 300 mm), sample pad (SB08, 15 × 300 mm), absorbent pad (H-1, 30 mm × 300 mm), immersing pad (8965, 10 mm × 300 mm), and nitrocellulose membrane (NC membrane, Sartorius CN 95, 25 × 300 mm) were obtained from Shanghai Jiening Biological Technology Co., Ltd. (Shanghai, China). All reagents were of analytical grade and used without any purification. The fluorescence reader used in this study was obtained from Suzhou Helmen Precision Instruments Co., Ltd. (Suzhou, China). The XYZ platform dispenser HM3035 and programmable strip cutter ZQ2002 were purchased from Shanghai Kinbio Tech Co., Ltd. (Shanghai, China).

#### 2.1.1. Preparation of the Conjugates of EuPSM and Antibodies

A measure of 100 μL of EuPSM was washed four times with MES buffer (50 mM, pH = 6.0). Then, 17.5 μL of EDC solution (100 mg/mL) and 17.5 μL of sulfo-NHS solution (100 mg/mL) were added, and the mixture was incubated on a shaker (37 °C, 220 rpm) for 45 min to activate the carboxyl groups. After washing the activated microspheres three times with MES buffer, 500 µL of MES buffer and the optimal antibody coating mass of anti-skTnI Ab2 was added to the mixture, which was then agitated for 2 h. The microspheres were then washed twice with ultrapure water and resuspended in 200 μL of blocking solution (5 μL/mL ethanolamine and 20 mg/mL BSA) for 30 min of continuous shaking. Finally, the microspheres were washed twice with ultrapure water and resuspended with 200 μL of ultrapure water to obtain the europium fluorescent microsphere–antibody complex and stored in the refrigerator at 4 °C.

#### 2.1.2. Preparation of EuPSM-Based LFIA

The EuPSM-based LFIA comprises an NC membrane, conjugate pad, sample pad, absorbent pad, and backing plate. Anti-skTnI Ab1, which recognizes the analyte, and DNP-BSA conjugates, which recognize the DNP antibody, were sprayed by an XYZ platform dispenser on the NC membrane to form the test line (T line) and control line (C line), respectively. Then, the mixture of EuPSM-anti-skTnI Ab2 conjugates and EuPSM-DNP Ab conjugates was sprayed onto the conjugate pad. The above-sprayed NC membrane and the conjugate pad were placed in a 37 °C oven for 2 h. After that, the conjugate pad was attached below the NC membrane, the absorbent pad was attached above the NC membrane, and the sample pad was attached below the conjugate pad, with a 2 mm overlap between adjacent parts. Finally, the assembled backing plate was cut using the programmable strip cutter and then put it into a reagent card box. For further quantitative analysis, the reagent card box was inserted into a portable fluorescence reader. It utilizes a UV excitation light source to scan the detection area point by point, outputting the fluorescence signals of the T line and C line.

### 2.2. Optimization of the LFIA

This study primarily optimized the detection sensitivity of EuPSM-LFIA through the following approaches: (1) the optimum size of europium fluorescent microspheres was screened. In this study, seven europium fluorescent microspheres, WD200, WD300, WD300+, JK191, JK288, BY200 and BY300, were used to select the most suitable europium fluorescent microspheres through sensitivity comparison experiments. (2) Pre-treatment of the conjugate pad using 200 nM Tris-HCl solution (containing 20% sucrose and 4% S9). (3) Optimization of the mass of anti-skTnI Ab2 (25, 50, 75, 100, and 150 μg) coated on EuPSM. (4) Optimization of the pH (pH 5, 6, 7, 8, and 9) of EuPSM solution to conjugate with anti-skTnI Ab2. (5) Optimization of the spraying concentration of the EuPSM-anti-skTnI Ab2 complex (2%, 4%, 6%, 8%, and 10%). (6) Optimization of the concentrations of Tween-20 and BSA in the sample diluent, with concentration gradients of 0.1%, 0.25%, 0.5%, 1%, 3%, and 5% of Tween-20; (7) 0%, 0.1%, 0.3%, 0.5%, 0.7%, and 0.9% of BSA, respectively. (8) Optimization of the pH of the sample diluent (5, 6, 7, 8, 9, and 10). (9) Optimization of the concentration of the antibody on the detection line (0.5, 1, 1.5, and 2 mg/mL). (10) Optimization of assay time to use the developed LFIA: 5 min, 10 min, 15 min, 20 min, and 30 min.

#### 2.2.1. Detection of Spiked Serum Samples

To verify the feasibility and accuracy of the optimized highly sensitive EuPSM-LFIA in clinical testing, normal human serum samples were selected as the detection matrix. Gradient concentrations of skTnI antigen standard solutions were prepared by sequentially adding the antigen to achieve final concentrations of 100, 50, 20, 10, 5, 2, 1, 0.5, 0.2, and 0 ng/mL in spiked serum samples. We also verified the specificity of the LFIA with known possible interfering species (i.e., CK-MB, CK-MM, cTnI, and Myo) in the clinical testing; the spiked concentration of four interfering biomarkers was 100 ng/mL. Then, 100 μL of each sample was applied to the sample pad in sequence. After an incubation period of approximately 15 min, a fluorescence reader was used to obtain the quantitative detection results.

#### 2.2.2. Evaluation of Detection Performance of EuPSM-LFIA

By detecting serum-spiked samples of skTnI, the performance of the optimized EuPSM-LFIA was evaluated in terms of detection linearity, detection limit, and detection precision.

#### 2.2.3. Evaluation of Detection Linearity

Human serum samples with gradient concentrations of skTnI were prepared and tested using the same batch of highly sensitive EuPSM-LFIA. Each sample was tested three times, and the quantitative detection results were read using a fluorescence reader. The average value of the three test results was taken as the detection signal value for each concentration. Origin 2021 software was used to plot a quantitative relationship curve between the antigen concentration in the skTnI human serum-spiked samples and the device detection signal value. The linear fitting coefficient of the curve was used as an evaluation index for the detection linearity of the device.

#### 2.2.4. Evaluation of the Detection Limit

Using the same batch of highly sensitive EuPSM-LFIA, skTnI serum-spiked samples with a zero-concentration calibrator were repeatedly tested 20 times. The quantitative detection results were read using a fluorescence reader, and the mean (M) and standard deviation (SD) of these 20 repeated measurements were calculated. According to the statistical determination index for the 95% confidence interval, the value of M + 3 × SD was used as the ordinate. This value was substituted into the formula of the above quantitative relationship curve to calculate the corresponding concentration, which served as the evaluation index for the lowest detection limit of the highly sensitive EuPSM-LFIA.

#### 2.2.5. Evaluation of Detection Precision

The skTnI serum-spiked samples with three calibration concentrations were measured three times, and the average detection signal intensity of each test strip was recorded. The mean ($\bar{x}$) and SD of the test results for each sample were calculated from three repeated measurements. The SD was divided by $\bar{x}$, and the result was multiplied by the percentage to obtain the coefficient of variation (CV), which was used to evaluate the detection precision of the highly sensitive EuPSM-LFIA.

## 3. Results and Discussion

### 3.1. Detection Principle of EuPSM-LFIA

**Figure 1:**
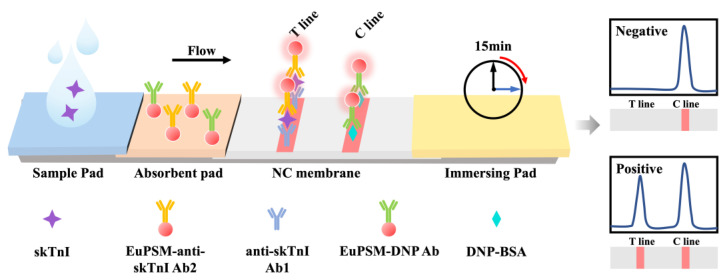
Schematic diagram of the EuPSM-LFIA.

As shown in Figure 1, the anti-skTnI Ab1 and DNP-BSA complex were immobilized on the NC membrane through hydrogen bonding, electrostatic adsorption, and hydrophobic interaction to form the T line and C line. In the presence of the target in the sample, the target is recognized by both the anti-skTnI Ab2 connected with EuPSMs and anti-skTnI Ab1 on the T line. Thus, the ‘sandwich’ structure of Ab1-target-Ab2 was formed, resulting in a color band with a clear fluorescence peak on the T line. The intensity of the fluorescence signal on the T line is positively correlated with the concentration of the target in the sample. Regardless of the presence of the target, the DNP-BSA complex on the C line can recognize the DNP antibody conjugated with the EuPSM, forming a visible band and a clear fluorescence peak on the C line, indicating that the platform is functioning normally.

### 3.2. Optimization of Sensitivity for EuPSM-LFIA

**Figure 2:**
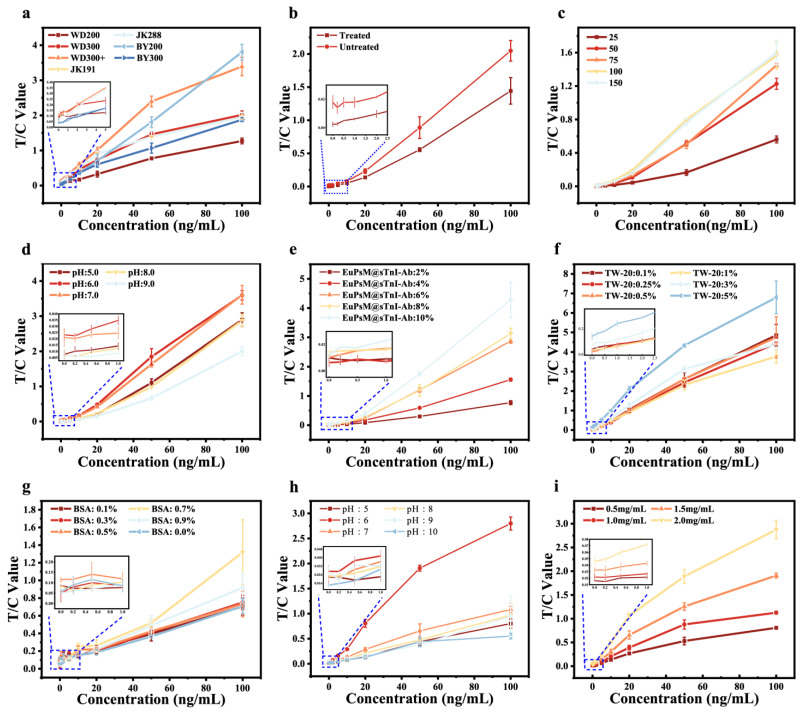
**Optimization of EuPSM-LFIA**. (**a**) EuPSM particle size screening. (**b**) Optimization results before and after pretreatment of the conjugate pad. (**c**) Antibody coating quality. (**d**) The pH for EuPSM-coupled antibody. (**e**) EuPSM@skTnI-Ab spraying concentration. The concentration of Tween-20 (**f**) and BSA (**g**). (**h**) The pH for sample dilution solution. (**i**) The concentration of cutting-edge membrane antibody.

From the working principle of the sensor, it can be seen that the final detection result is directly related to the presence and depth of the fluorescence signal on the detection line, and the fluorescence signal is the signal of europium fluorescent microspheres gathered on the detection line. Therefore, the particle size of europium fluorescent microspheres is important for the strength of the fluorescence signal. As shown in Figure 2a, seven europium fluorescent microspheres, namely WD200 (200 nm), WD300 (290 nm), WD300+ (296 nm), JK191 (191 nm), JK288 (288 nm), BY200 (220 nm) and BY300 (330 nm), were, respectively, used in this study to carry out experiments. It was found that the detection signal-to-noise ratio (SNR) of europium fluorescent microspheres of WD300+ was superior to other microspheres in both high and low concentrations.

The conjugate pad is primarily composed of glass fiber, which serves as a crucial component for storing the complex of EuPSM-anti-skTnI Ab2. During the operation of the LFIA, it is essential to release this complex from the conjugate pad. To ensure full and complete release of the complex, the conjugate pad was treated using Tris-HCl solution containing 20% sucrose and 4% S9. As shown in Figure 2b, the pretreated conjugate pad demonstrated a controlled-release effect on the complex of the EuPSM-anti-skTnI Ab2. This is because the sucrose in the pretreatment solution can protect the activity of the complex of EuPSM-anti-skTnI Ab2, ensuring the sensitivity and specificity of the LFIA. The surfactant S9 in the pretreatment solution can reduce the surface tension of the sample solution, allowing the sample to more easily penetrate and the complex of EuPSM-anti-skTnI Ab2 to more completely release, which promoted more thorough binding between the antigen and the antibody, thereby enhancing the overall detection signal.

As shown in Figure 2c, the quality of EuPSM-anti-skTnI Ab2 was optimized in this study. The detection results indicated that when the antibody coating mass was within the range of 25–100 μg, the detection signal value increased with the coating mass. However, when the coating mass increased to 150 μg, there was no significant difference in the detection signal value compared to 100 μg. Considering cost-effectiveness, we selected 100 μg as the optimal antibody coating mass for EuPSMs in this study.

As shown in Figure 2d, the optimal pH value for coupling antibodies to EuPSMs was determined in this study. The test results indicated that at pH 6 and 7, there was a noticeable improvement in the overall detection signal value of the LFIA. However, at pH 6, the distinction between low concentrations was more pronounced. So, pH 6 was selected as the optimal pH for coupling antibodies to EuPSMs.

Following the optimization of antibody coating quality and coupling pH for EuPSMs, the optimal spray concentration of EuPSM–antibody complex on the conjugate pad was further optimized in this study. The test results, as shown in Figure 2e, revealed that the overall detection signal value of the LFIA increased with higher spray concentrations. However, analysis of low-concentration test results showed that spray concentrations of 8% and 10% exhibited poorer distinction and linearity for low concentration samples, whereas a spray concentration of 6% demonstrated better linearity and distinction. Therefore, a 6% spray concentration was selected as optimal for the EuPSM–antibody complexes.

The optimization of the Tween-20 and BSA content in the sample diluent was investigated in this study. As shown in Figure 2f, a Tween-20 content of 5% yielded significantly superior overall detection signal compared to other concentrations. Additionally, a BSA content of 0.5% (Figure 2g) resulted in higher distinction for low concentrations, contributing to improved detection sensitivity. Therefore, 5% Tween-20 and 0.5% BSA were identified as the optimal component concentrations for the sample diluent.

As illustrated in Figure 2h, the optimal pH of the sample diluent was optimized in this study. The test results showed that at pH 6, the overall detection signal value of the LFIA surpassed that of other pH values, making it the preferred choice for the sample diluent.

As shown in Figure 2i, the concentration of the detecting antibody on the T line was optimized in this study. The detection results demonstrated that an increase in the concentration of membrane-coated antibodies corresponded to an increase in the detection signal value of the LFIA. This increase was attributed to the capture of more EuPSM–antibody complexes at the test line, resulting in an enhanced detection signal and improved detection sensitivity. However, a continued increase in the concentration of the detecting antibody posed a risk of heightened non-specific adsorption at the test line, leading to false positive results, potentially leading to false-positive results. Additionally, considering both performance and cost-effectiveness, a concentration of 1.5 mg/mL was selected as the optimal coating concentration for the detecting antibody on the detection line.

To figure out the optimal assay time, we conducted experiments to evaluate LFIA detection performance at different assay times: 5 min, 10 min, 15 min, 20 min, and 30 min. As shown in Figure S1, a shorter assay time resulted in high background fluorescence and a relative higher coefficient of variation (CV). When the assay time was 15 min or later, the T/C value was kept relative stability while the fluorescence intensity of both the T line and C line became weaker due to fluorescence attenuation. Therefore, 15 min was the optimal assay time for LFIA since it provided the best signal in a relatively shorter time.

### 3.3. Detection Feasibility and Performance of Serum-Spiked Samples

#### 3.3.1. Detection Feasibility of Serum-Spiked Samples

**Figure 3:**
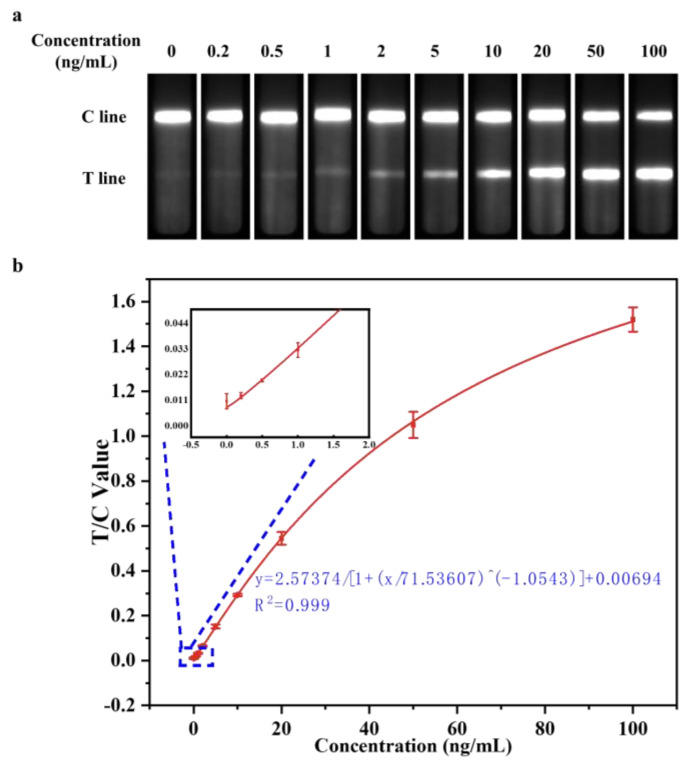
**Detection of skTnI-spiked serum samples by EuPSM-LFIA**. (**a**) Optimized biosensor detection results for serum addition samples. (**b**) Quantitative standard curves for portable muscle injury instant-detection sensors.

**Figure 4:**
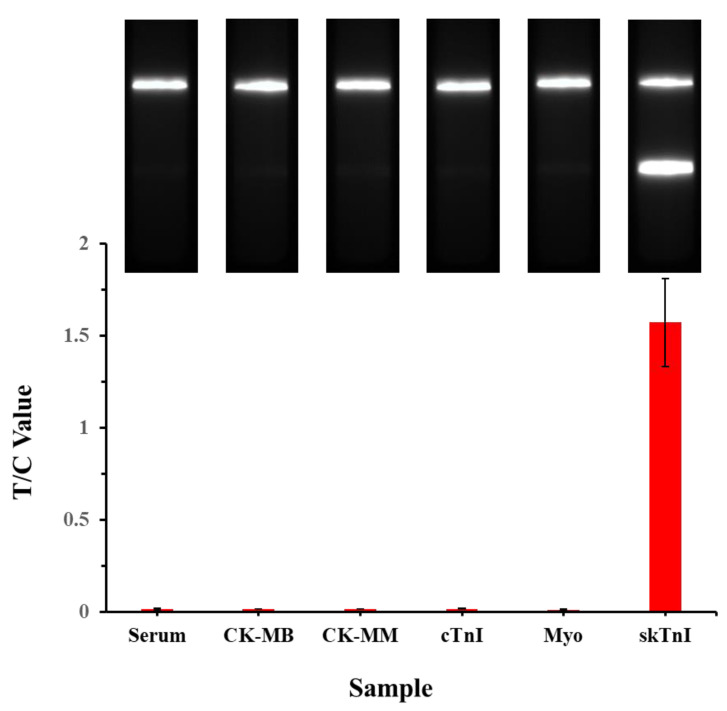
**Evaluation of the specificity of the optimized LFIA**.

To verify the feasibility and accuracy of the highly sensitive muscle injury immediate detection device optimized in the above experiments in a clinical testing, negative human serum samples and sample dilutions were selected as the detection matrix. SkTnI antigen standards were added in sequence, and gradient concentrations for 100, 50, 20, 10, 5, 2, 1, 0.5, 0.2, and 0 ng/mL of serum-spiked were prepared. The optimized EuPSM-LFIA was then employed to detect the prepared skTnI serum-spiked samples, with the test results shown in Figure 3a. Notably, when the concentrations of the skTnI serum-spiked samples were 0 and 0.2 ng/mL, respectively, there was no significant difference in signal intensity observed by the naked eye. However, when the concentration of skTnI serum-spiked sample reached 0.5 ng/mL, there was a noticeable difference in signal intensity compared to the 0 ng/mL sample, indicating a positive result. This suggests that the optimized device can achieve a detection sensitivity of 0.5 ng/mL for the skTnI serum-spiked samples, with a concentration of 0.5 ng/mL serving as the exclusion threshold for clinical judgment of muscle injury [13,18]. In addition, compared to the negative detection results for four interfering species and the negative control, the LFIA tested positive for skTnI detection, indicating that the optimized LFIA has good specificity (Figure 4). Therefore, the optimized EuPSM-LFIA can effectively determine whether a subject has a muscle injury, enabling instant detection.

#### 3.3.2. Evaluation of Detection Linearity

Using the concentration of skTnI in serum-spiked samples (100, 50, 20, 10, 5, 2, 1, 0.5, 0.2, and 0 ng/mL) as the *x*-axis and the mean the detection signal intensity after three repeated measurements of each concentration as the *y*-axis, the quantitative curve for the detection of skTnI serum-spiked samples by the optimized highly sensitive EuPSM-LFIA was plotted, as shown in Figure 3b. The four-parameter fitting relationship of the quantitative curve was *y* = 2.57374/[1 + (*x*/71.53607)^(−1.0543)] + 0.00694, indicating that the detection signal intensity of the device was positively correlated with the concentration of the target in the sample, meaning that the detection signal intensity increases with the concentration of the target substance in the sample. The correlation coefficient *R*^2^ = 0.999, demonstrating a strong quantitative relationship between the detection signal intensity and the concentration of the target substance in the serum-spiked sample. Therefore, the optimized highly sensitive EuPSM-LFIA meets the standard detection range.

#### 3.3.3. Evaluation of Detection Limit

After conducting 20 repeated measurements on the zero-concentration calibrator of skTnI serum-spiked samples, the M and SD of the detection signal intensity of the device were calculated to be M = 0.012544 and SD = 0.002881 and M + 3 × SD = 0.021187, respectively. According to the statistical determination index of a 95% confidence interval, M + 3×SD was substituted as y into the above quantitative curve *y* = 2.57374/[1 + (*x*/71.53607)^(−1.0543)] + 0.00694, yielding *x* = 0.520405. Therefore, the LOD of the optimized highly sensitive EuPSM-LFIA was approximately 0.5 ng/mL.

#### 3.3.4. Evaluation of Detection Precision

In this study, the detection precision of a highly sensitive EuPSM-LFIA was evaluated through the CV. The skTnI serum-spiked samples with calibrated concentrations of 30, 50, and 75 ng/mL were measured three times. The average detection signal intensity was found to be 1.46231, 1.29458, and 0.90577, respectively. The variation in the above three concentrations was calculated to be 2.99%, 7.16%, and 10.38%, respectively. The average CV for these three concentrations was 6.84%, indicating that the optimized highly sensitive EuPSM-LFIA had a low CV and good detection precision, meeting the requirement of CV < 15% specified in the detection standard.

## 4. Conclusions

In this paper, a highly sensitive EuPSM-LFIA was successfully developed. The device achieves high performance mainly through the following approaches: optimizing the pre-treatment method of the conjugate pad; adjusting the coupling pH value of fluorescent nanospheres and antibodies; improving the detection concentration of fluorescent nanosphere–antibody complexes; and adjusting the concentration of sample diluent components. With these optimized improvements, the developed muscle injury detection device can achieve highly sensitive detection of 0.5 ng/mL skTnI within 15 min. Compared to traditional gold nanoparticle test strips, the sensitivity of this method is relatively higher. However, compared to ELISA and chemiluminescence, both the detection range and detection limit are inferior. In this study, we developed the first LFIA specifically for monitoring muscle injuries caused by exercise at the point of care, demonstrating that it is both sufficient and suitable for this purpose. We are continuing this research by further improving its detection limit. Furthermore, after preliminary experimental evaluations, the device showed excellent detection performance, with a linear correlation coefficient of *R*^2^ reaching 0.999, a minimum detection limit as low as 0.5 ng/mL, and an average CV of 6.84%. These results show that the developed EuPSM-LFIA possesses high sensitivity and excellent detection performance, meeting the requirements of clinical detection. It provides a novel approach for the timely detection, early diagnosis, and long-term monitoring of muscle injuries.

## Data Availability

Data are contained within the article.

## Supplementary Materials

The following supporting information can be downloaded at: https://www.mdpi.com/article/10.3390/bios14080381/s1, Figure S1: LFIA detection performance at different assay time.
