# Supplementary material for: A Fluorescent Lateral Flow Immunoassay for the Detection of Skeletal Muscle Troponin I in Serum for Muscle Injury Monitoring at the Point of Care
Source: Biosensors (Basel). 2024 Aug 8;14(8):381. doi: 10.3390/bios14080381 (PMC11352793; doi:10.3390/bios14080381)
Supplement: Supplementary file 1 [file biosensors-14-00381-s001.zip › biosensors-3087830-supplementary.pdf]

biosensors

MDPI

Communication

A Fluorescent Lateral Flow Immunoassay for Detection of Skeletal Muscle Troponin I in Serum for Muscle Injury Monitoring at Point-of-Care

Deding Tang [1,2,3], Shuang Wu [1,2], Mengqi Kong [1,2,5], Zhaonan Liu [1,2,5], Zonghao Li [1,2,5], Ying Han [4], Yan Gong [4,*] and Jie Hu [5,*]

[1]The Key Laboratory of Biomedical Information Engineering of Ministry of Education, School of Life Science and Technology, Xi'an Jiaotong University, 710049, Xi'an
[2]Bioinspired Engineering and Biomechanics Center (BEBC), Xi'an Jiaotong University, 710049, Xi'an
[3]Maanshan Teacher's College, 243041, Maanshan
[4]School of Chemistry and Life Sciences, Suzhou University of Science and Technology, 215009, Suzhou
[5]Suzhou Diyinan Biotech Company, 215129, Suzhou
*Corresponding authors: gongyan@usts.edu.cn, jason.hu@sz-dna.cn

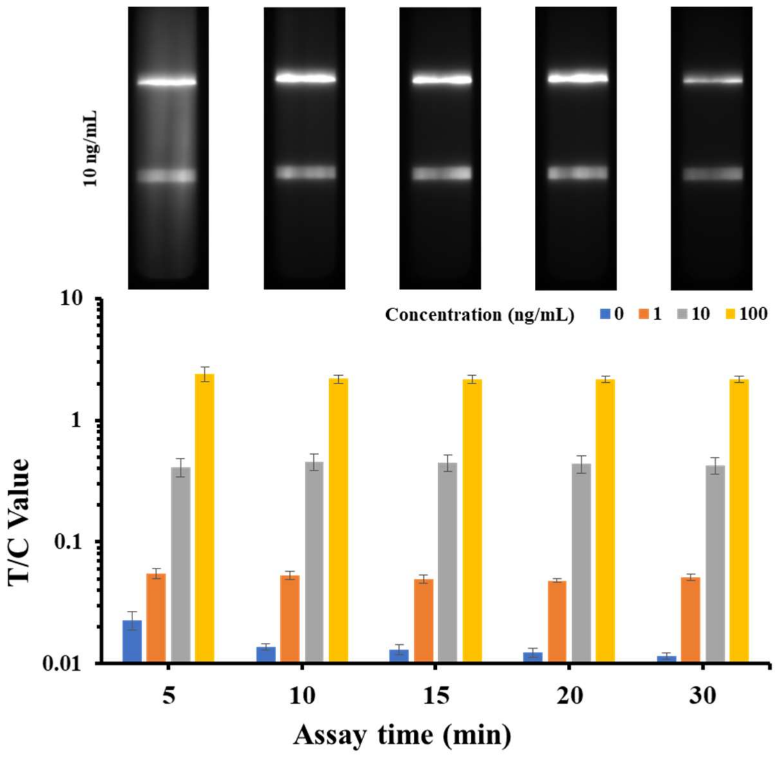

Fig. S1 LFIA detection performance at different assay time
